# PALB2 as a factor to predict the prognosis of patients with skull base chordoma

**DOI:** 10.3389/fonc.2022.996892

**Published:** 2022-09-08

**Authors:** Yujia Xiong, Mingxuan Li, Yutao Shen, Tianshun Ma, Jiwei Bai, Yazhuo Zhang

**Affiliations:** ^1^ Beijing Neurosurgical Institute, Capital Medical University, Beijing, China; ^2^ Department of Neurosurgery, Beijing Tiantan Hospital, Capital Medical University, Beijing, China; ^3^ Beijing Institute for Brain Disorders Brain Tumor Center, Beijing, China; ^4^ China National Clinical Research Center for Neurological Diseases, Beijing, China

**Keywords:** chordoma, PALB2, prognosis, immunohistochemistry, nomogram

## Abstract

**Objective:**

This study aimed to study the role of PALB2 on the prognosis of skull base chordoma patients and the proliferation, migration, and invasion of chordoma cells.

**Methods:**

187 patients with primary skull base chordoma were involved in the study. Immunohistochemical analysis was used to measure the PALB2 protein expression. Kaplan-Meier analysis, univariate and multivariate Cox analysis were used to evaluate the impact of PALB2 on patient prognosis. A nomogram was established for predicting the progression free survival of chordoma patients. Cell counting kit-8, colony formation, transwell migration, and invasion assays were used to assess the proliferation, migration, and invasion of chordoma cells with PALB2 knockdown. TIMER 2.0 was used to explore the expression and prognostic role of PALB2 in cancers.

**Results:**

High PALB2 expression indicated an adverse prognosis in chordoma. A nomogram involved PALB2, degree of resection, pathology, and Al-mefty classification could accurately predict the progression free survival of chordoma patients. The proliferation, migration, and invasion of chordoma cells significantly decreased after PALB2 knockdown. Additionally, PALB2 showed high expression in various cancers and was associated with a poor prognosis.

**Conclusion:**

In summary, our results reveal that high PALB2 expression indicates a poor prognosis of chordoma patients and promotes the malignant phenotypes of chordoma cells *in vitro*.

## Introduction

Chordomas are rare bone tumors located in the skull base and axial skeleton (1). The incidence of chordomas is approximately 5/1000,000 (2). So far, surgical resection combined with radiotherapy is still the preferred treatment for chordomas (3). There is no approved drug for the treatment of chordomas (4). Chordomas are often adjacent to important structures, so it is difficult to remove tumors completely, which makes chordomas prone to recurrent (5). It is important to identify effective prognostic markers and explore the underlying mechanism of chordoma.

Partner and Localizer of BRCA2(PALB2) is a protein-coding gene located in chromosome 16p12.2. Robust data demonstrates that PALB2 mainly participates in DNA homologous recombination repair by binding to BRCA2 (6, 7). Recent studies reveal that the pathogenic variants of PALB2 are associated with some cancers, such as breast, pancreatic, and ovarian cancers (8, 9). PALB2 mutations lead to increased susceptibility to these tumors. In addition, high PALB2 expression leads to a poor prognosis in advanced breast cancer (10). However, to date, the role of PALB2 in chordoma remains to be studied.

In our study, we investigated the potential prognostic role of PALB2 in skull base chordomas, and a nomogram was further constructed. Moreover, we explored the effect of PALB2 on chordoma cells using several cellular experiments *in vitro*. In addition, we analyzed the possible role of PALB2 in other tumors.

## Methods

### Patients

A total of 187 patients with primary skull base chordoma received surgical treatment in the Neurosurgery Department of Beijing Tiantan Hospital from January 2008 to December 2014 were included in the study. Patients were included according to the following criteria (1) the pathological diagnosis was chordoma (2), complete clinical information (3), the tumor was located at the skull base. Exclusion criteria (1) tumor was too few to build the tissue microarray (TMA) (2) patients had other malignant tumors (3) patients were lost to follow up.

### Clinical information

Tumor volume was estimated based on preoperative and intraoperative findings (length × width × height/2). We divided chordomas into tree types using AL-Mefty classification (11). Type I: the tumor was confined to a single anatomic space at the skull base. Type II: the tumor invaded 2 or even more anatomic spaces at the skull base, but the tumor could be completely removed *via* one surgical approach. Type III: the tumor extensively invaded multiple anatomic spaces, and the tumor couldn’t be completely removed through one surgical approach. Preoperative and postoperative imaging were compared to estimate the extent of surgical resection (12). Total resection (TR): postoperative imaging showed no residual tumor; subtotal resection (STR): residual tumor ≤5%; and partial resection (PR): postoperative imaging showed residual tumor >5%. Rich blood supply: tumor resection surface bled easily and was difficult to aspirate clearly; poor blood supply: tumor resection surface bled less and was easy to aspirate clearly.

### Immunohistochemistry

Chordoma tissues from the 187 chordoma patients were fixed with formalin and embedded in paraffin for following TMA construction. After assayed by TMA using the Tissue Array MiniCore (ALPHELYS, Plaisir), the most representative parts of the tissue were selected to build the TMA. Then TMAs were cut into 4μm thick sections for further staining with Leica RM 2135 Rotary Microtome (Rankin, Wetzlar, Germany). Leica BOND III automated system was used to perform Immunohistochemical staining with primary antibody (anti-PALB2 antibody, ab-202970, abcam, 1:500) and Bond Polymer Refine Detection (Leica Biosystems, DS9800) including secondary antibody. Leica Aperio AT2 scanner was used to scan TMA images at 400× magnification. Three pathologists examined the slides independently. Then, we analyzed the images by Leica Aperio ImageScope software (version 12.3). The H-score was used to evaluate staining intensity. H-Score (12) = 1 × (percent of light staining cells) + 2 × (percent of moderate staining cells) + 3 × (percent of strong staining cells).

### Cell culture, cell counting kit-8, cell migration and invasion, colony formation assays

MUG-Chor1 and UM-Chor1 chordoma cell lines, donated by the Chordoma Foundation (for MUG-Chor1) and purchased from ATCC (for UM-Chor1), were cultured in IMDM (Isocove’s modified Dulbecco’s medium)/1640-RPMI (4:1) with 10% fetal bovine serum in an incubator at 37°C and 5% CO_2_.

The small interfering RNAs (siRNAs) (siPALB2-1: GCAGTGAACTTACTACTCA, siPALB2-2: GGACCTTATTGTTCTACCA, siPALB2-3: GAACTCACCTACAATAACT), purchased from RiboBio Medical Biotechnology (Guangzhou, China), were used to silence PALB2 in chordoma cell lines with lipofectamine 3000 (Invitrogen). After seeding UM-Chor1 cells and MUG-Chor1 cells into plates, PALB2 siRNA (si-PALB2) or control siRNA (si-NC) was transfected to the cells (concentration: 50nM).

After culturing chordoma cells in the 96-well plate (MUG-Chor1:6×10^3^) cells/well, UM-Chor1: 2.5×10^3^ cells/well) for 24 hours, chordoma cells were treated with si-PALB2 or si-NC. The optical density (OD) values at 450 nm were measured at 24, 48, 72, 96 hours, respectively after 2 hours incubation of CCK-8 (Dojindo).

For migration and invasion assays, 1×10^5^ MUG-Chor1 cells and 2.5×10^4^ UM-Chor1 cells transfected with si-PALB2 or si-NC were added into the transwell chamber with (invasion) or without (migration) Matrigel. After 48 hours, the cells on the surface were fixed, then stained with 0.1% crystal violet.

For colony formation assay, 2×10^3^ chordoma cells transfected with si-PALB2 or si-NC were incubated into 6-well plates and cultured at 37°C for 2 weeks. Cell colonies were fixed and stained with 0.1% crystal violet.

### Western blot and quantitative reverse transcription polymerase chain reaction

After transfection for 2 days, chordoma cells were collected for proteins and RNA extraction. For western blot, we used 10% sodium dodecyl sulfate-polyacrylamide gel electrophoresis to separated protein samples (20 μg). Then, protein blots were transferred to polyvinylidene difluoride membranes. The bands were incubated overnight with anti-PALB2 (ab202970) and anti-GAPDH or anti-β-actin (ZSGB-BIO) antibodies at 4°C, followed by secondary antibodies. Chemiluminescence was performed by Amersham Imager 600 for blot visualization.

After RNA extraction, the SuperScript III First-Strand Synthesis System (Invitrogen) was used to synthesize cDNA. QRT-PCR was performed using the QuantStudio 5 (Applied Biosystems). The expression of PALB2 was normalized according to GAPDH. The primers were as follows: GAPDH: 5’-GGAGCGAGATCCCTCCAAAAT-3’ (Forward) and 5’-GGCTGTTGTCATACTTCTCATGG-3’ (Reverse), PALB2: 5’- AGGGAATACAGCAAGACACTAG-3’ (Forward) and 5’-GATCCTGCTGAGACAAACAATC-3’ (Reverse).

### Pan-cancer analysis

TIMER 2.0 was used to analyze the difference in PALB2 expression between normal tissue and tumor tissue in 33 types of cancer. The Kaplan-Meier analysis was used to explore the impact of PALB2 on the prognosis of liver hepatocellular carcinoma (LIHC) and lower-grade glioma (LGG) patients *via* the GEPIA website. Chordoma RNA sequencing datasets were obtained from dbGaP phs002301.v1.p1. LIMMA R package was used to select genes associated with PALB2. The clusterProfiler R package and enrichplot R package were applied to perform the KEGG enrichment analysis.

### Statistical analysis

Statistical analysis was performed using R software (version 3.6.3) and SPSS (version 22.0, IBM Corp). The relationship between PALB2 and clinical characters was analyzed using Wilcoxon Mann–Whitney test, χ2 test, and 2 independent samples t-test. Survival R package and survminer R package were used to perform Kaplan-Meier analysis, Proportional Hazard assumption, univariate and multivariate Cox analysis. The nomogram and calibration curve were drawn using R software with rm, Hmisc, lattice, survival, ormula, and ggplot2 packages. And survivalROC package was used to draw the ROC curve. *P* value <0.05 indicated statistical significance.

## Results

### Associations between PALB2 and clinical characters in chordoma

187 primary skull base chordoma patients were involved in this study (**Table 1**; **Supplementary Table 1**). Those patients were separated into 2 groups by the median of the H-score of PALB2 (101.0). In addition, we analyzed the correlations between PALB2 and clinical characteristics. There was no significant difference in the distribution of males and females in the two groups (*P*=0.83). The mean age of patients was 39 years (range 3-78 years) in the high PALB2 group and 41 years (range 8-76 years) in the other group (*P*=0.45). The course of the disease ranged from 1 month to 84 months (mean 13 months) in the high group and 1 month to 360 months (mean 27 months) in the low group (*P*=0.06). The mean tumor volume in the high group was 34.7 cm^3^ (range 1.7-258.0 cm^3^), while in the low group, the mean tumor volume was 28.7 cm^3^ (range 2.5-152.0cm^3^) (*P*=0.19). Interestingly, we found that there were more conventional chordomas in the high PALB2 group (*P*=0.02). The distribution of Al-Mefty classification in the two groups showed no difference (*P*=0.46). Moreover, there was no remarkable difference in the proportion of patients who underwent endoscopy or craniotomy between the two groups (*P*=0.14). 59/94 patients received TR/STR in the high PALB2 group and 66/93 patients in the low PALB2 group (*P*=0.28). For blood supply, the tumor blood supply was rich in 109 patients and poor in 78 patients, no significant difference was observed between the two groups (*P*=0.51). By the end of follow-up, 129 patients suffered tumor recurrence and 72 patients died.

**Table 1:** Patient characteristics.

Characteristic	Total	PALB2 expression		P value
		High	Low	
Gender				0.83
Male	98	50	48	
Female	89	44	45	
Age, mean (range), year	40 (3-78)	39 (3-78)	41 (8-76)	0.45
Course of disease, mean (range), month	20 (1-360)	13 (1-84)	27 (1-360)	0.06
Tumor volume in cm^3^, mean (range)	31.7 (1.7-258.0)	34.7 (1.7-258.0)	28.7 (2.5-152.0)	0.19
Pathology				0.02
Conventional	126	71	55	
Chondroid	61	23	38	
AL-mefty				0.46
Type I	34	15	19	
Type II	89	43	46	
Type III	64	36	28	
Surgical approach				0.14
Endoscopic transsphenoidal	79	45	34	
Craniotomy	108	49	59	
Extent of resection				0.28
Total resection/Subtotal resection	125	59	66	
Partial resection	62	35	27	
Blood supply				0.51
Poor	78	37	41	
Rich	109	57	52	

### High levels of PALB2 indicated an adverse prognosis

**Figure 1:**
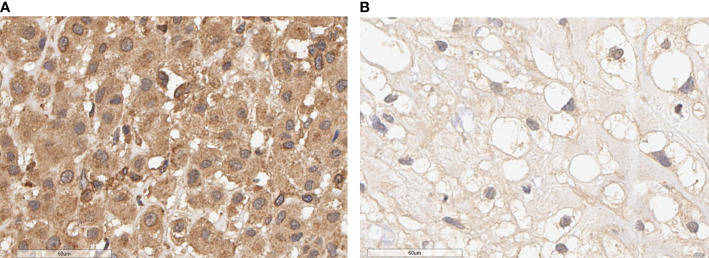
PALB2 immunohistochemical image of skull base chordoma (×400). **(A)** High expression of PALB2. **(B)** Low expression of PALB2.

**Figure 2:**
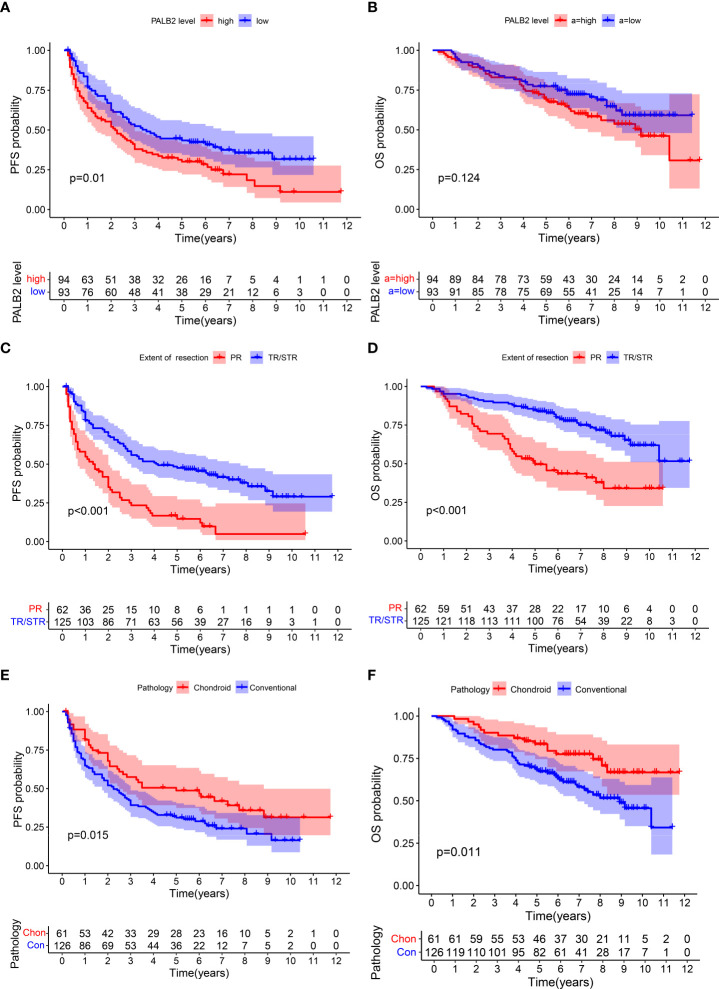
Kaplan–Meier survival curves of PFS and OS. PFS: **(A)** PALB2, **(C)** extent of resection, **(E)** pathology. OS: **(B)** PALB2, **(D)** extent of resection, **(F)** pathology.

The expression of PALB2 in 187 human chordoma tissues was analyzed by immunohistochemical staining, and we found that PALB2 was variedly expressed between chordoma patients, with the H-scores range from 1.74 to 177.5 (**Figure 1**). Then, we performed the Kaplan-Meier survival analysis between the low and high PALB2 expression groups, and our data suggested that the progression-free survival (PFS) times of patients with a high level of PALB2 were significantly shorter than those of patients with low PALB2 (**Figure 2A**). However, PALB2 has no significant effect on OS (p=0.124, **Figure 2B**). In addition, we found that the extent of resection, pathology, tumor volume, blood supply of tumor, and AL-Mefty level could impact both the OS and PFS of patients (**Figure 2C–F**; **Supplementary Figure 1**). The course of the disease was correlated with OS but not PFS (**Supplementary Figure 1**). Age, gender, and tumor calcification were not associated with prognosis (**Table 2**).

**Table 2:** Kaplan-Meier survival analysis of clinical characters.

Variables	P value	
	PFS	OS
PALB2	0.01	0.124
Extent of resection	<0.001	<0.001
pathology	0.015	0.011
tumor volume	0.002	0.044
blood supply	0.028	0.011
course of the disease	0.645	0.02
Age	0.485	0.708
gender	0.68	0.916
tumor calcification	0.687	0.332

**Figure 3:**
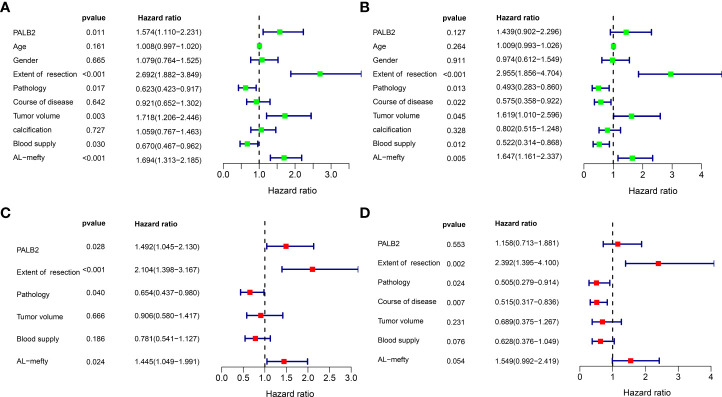
Univariate Cox and Multivariate Cox analysis of PFS and OS. **(A)** Univariate Cox analysis of PFS. **(B)** Univariate Cox analysis of OS. **(C)** Multivariate Cox analysis of PFS. **(D)** Multivariate Cox analysis of OS.

Univariate Cox(All the variables satisfy the PH assumption) revealed that high PALB2, partial resection, conventional chordoma, large tumor volume, the rich blood supply of the tumor, and high Al-mefty classification were risk factors for PFS (**Figure 3A**). And partial resection, conventional chordoma, the long course of the disease, large tumor volume, the rich blood supply, and high AL-mefty classification were risk factors for OS (**Figure 3B**). Moreover, multivariate Cox revealed that PALB2, the extent of resection, pathology, the course of the disease, and Al-mefty were independently associated with prognosis in skull base chordoma (**Figures 3C, D**).

### A nomogram for individual PFS prediction in skull base chordoma

**Figure 4:**
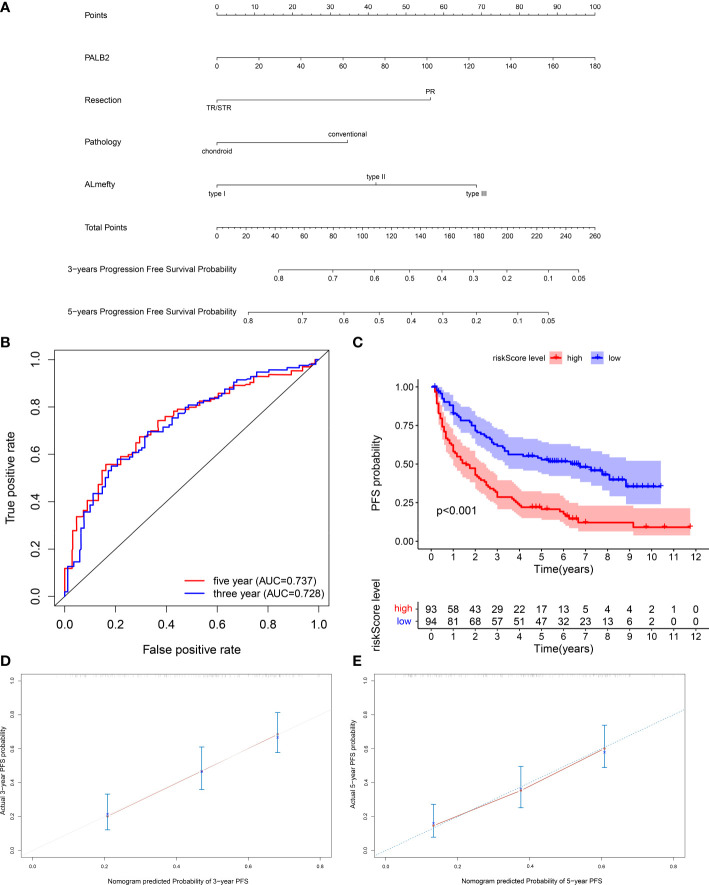
**(A)** Nomogram of 3-year and 5-year PFS in skull base chordoma patients. **(B)** ROC curves of the nomogram. **(C)** Kaplan–Meier survival curve of the patients separated by nomogram-predicted score. **(D)** Calibration curve of the nomogram prediction of 3-year PFS. **(E)** Calibration curve of the nomogram prediction of 5-year PFS.

Based on the results of univariate Cox and multivariate Cox analysis, we selected PALB2, degree of resection, pathology, and AL-mefty classification to establish a nomogram model to predict the PFS of skull base chordoma patients (**Figure 4A**). The ROC curve revealed that the nomogram showed adequate performance in the 3-year (AUC=0.728) and 5-year (AUC=0.737) PFS prediction (**Figure 4B**). Moreover, patients were divided into two groups according to the median riskscore calculated by the nomogram. The PFS times of patients with high riskscore were shorter than those with low riskscore (**Figure 4C**). Then, bootstrap was used to select samples for verifying the accuracy of the nomogram model (62 samples/time, 1000 times). The calibration curve showed the model properly predicted 3-year PFS and 5-year PFS (**Figures 4D, E**).

### Knockdown of PALB2 suppressed chordoma cells proliferation, migration, and invasion

**Figure 5:**
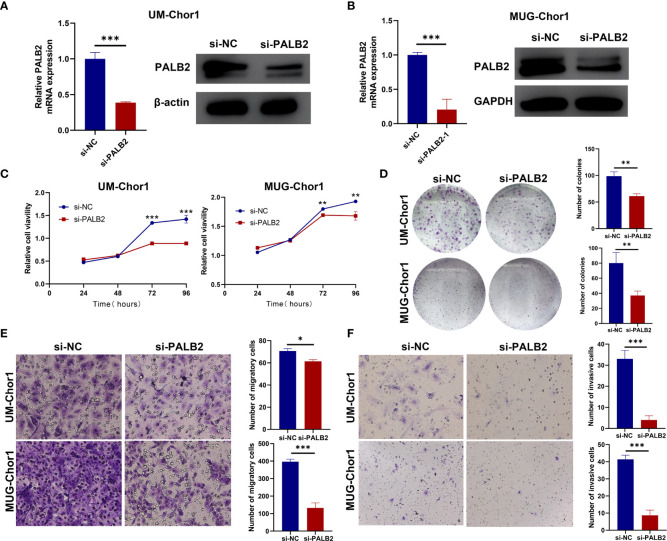
The function of PALB2 in chordoma cell lines. **(A)** QRT-PCR (left) and western blotting (right) analysis showed that the expression of PALB2 was decreased after being transfected with si-PALB2 in UM-Chor1. **(B)** QRT-PCR (left) and western blotting (right) analysis showed that the expression of PALB2 was decreased after transfected with si-PALB2 in MUG-Chor1. CCK8 assay **(C)** and Colony Formation Assays **(D)** indicated that the proliferation ability of chordoma cells (UM-Chor1 and MUG-Chor1) was decreased after being treated by si-PALB2. Transwell assay revealed that knockdown PALB2 inhibited the migration **(E)** and invasion **(F)** ability of UM-Chor1 and MUG-Chor1 chordoma cells. The results represent the mean ± s.d. of three independent experiments. Student’s t test **(A–F)**. *p < 0.05, **p < 0.01, ***p < 0.001.

The effect of PALB2 in chordoma cells was assessed in both UM-Chor1 and MUG-Chor1. We first confirmed reduced PALB2 expression after transfection of si-PALB2 using qRT-PCR and western blot (*P*<0.001, **Figures 5A, B**). CCK-8 was then used to measure cell viability. The proliferation of chordoma cells significantly reduced at 72 hours and 96 hours after si-PALB2 transfection compared to the si-NC group (**Figure 5C**). Colony formation experiments also showed that cells with PALB2 knockdown had fewer colonies (**Figure 5D**). The transwell assay revealed that the number of migrating and invading cells notably decreased after PALB2 knockdown (**Figures 5E, F**).

### High PALB2 predicted poor prognosis in several cancers

**Figure 6:**
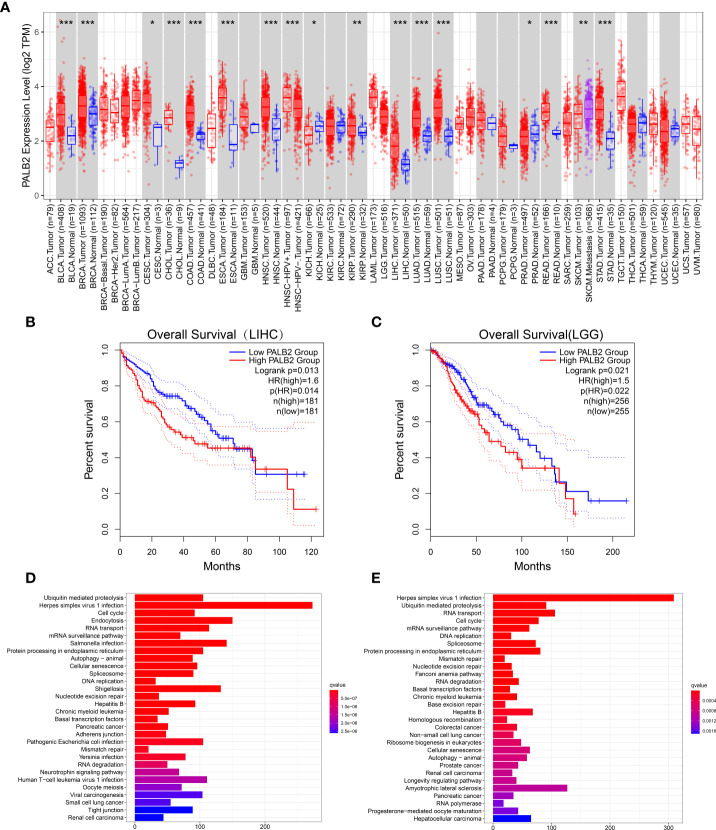
**(A)** Different PALB2 expression levels between tumor and normal tissue from TCGA. (adrenocortical carcinoma: ACC, bladder urothelial carcinoma: BLCA; breast invasive carcinoma: BRCA; cervical squamous cell carcinoma: CESC; cholangiocarcinoma: CHOL; colon adenocarcinoma: COAD; lymphoid neoplasm diffuse large B cell lymphoma: DLBC; esophageal carcinoma: ESCA; glioblastoma multiforme: GBM; brain lower grade glioma: LGG; head and neck squamous cell carcinoma: HNSC; kidney chromophobe: KICH; kidney renal clear cell carcinoma: KIRC; kidney renal papillary cell carcinoma: KIRP; acute myeloid leukemia: LAML; liver hepatocellular carcinoma: LIHC; lung adenocarcinoma: LUAD; lung squamous cell carcinoma: LUSC; mesothelioma: MESO; ovarian serous cystadenocarcinoma: OV; pancreatic adenocarcinoma: PAAD; pheochromocytoma and paraganglioma: PCPG; prostate adenocarcinoma: PRAD; rectum adenocarcinoma: READ; sarcoma: SARC; skin cutaneous melanoma: SKCM; stomach adenocarcinoma: STAD; testicular germ cell tumors: TGCT; thyroid carcinoma: THCA; thymoma: THYM; uterine corpus endometrial carcinoma: UCEC; uterine carcinosarcoma: UCS; and uveal melanoma: UVM). Kaplan–Meier survival curve showed that high PALB2 expression indicated a poor prognosis in LIHC **(B)** and LGG (**C**). KEGG pathways analysis of genes strongly associated with PALB2 in LIHC **(D)** and LGG **(E)**. *p < 0.05, **p < 0.01, ***p < 0.001.

To further explore the role of PALB2 in cancers, we performed a pan-cancer analysis of PALB2 in 33 types of cancers. Interestingly, the PALB2 expression level was higher in more than 10 types of cancers than that in the normal controls (**Figure 6A**). Moreover, we found that high expression of PALB2 indicated a poor prognosis in LIHC and LGG (**Figures 6B, C**). We then performed KEGG enrichment analysis and genes significantly correlated (cor>0.3, *P*<0.05) with PALB2 in LIHC and LGG were applied. The KEGG results showed PALB2 was associated with several cancer-associated pathways, including ubiquitin-mediated proteolysis, protein processing in the endoplasmic reticulum, autophagy, DNA replication, RNA transport, and cell cycle (**Figures 6D, E**). In addition, We perform KEGG enrichment analysis for genes significantly correlated (cor>0.3, *P*<0.05) with PALB2 in chordoma. Enrichment results showed that PALB2 may affect the Cell cycle and DNA replication in skull base chordoma, which is similar to LIHC and LGG. Interestingly, PALB2 may also affect many immune-related pathways, such as Th17 cell differentiation, the Chemokine signaling pathway, PD-L1 expression, PD-1 checkpoint pathway in cancer, and so on(**Supplementary Figure 2**).

## Discussion

Chordoma is easy to relapse, with a 5-year recurrent rate of about 59.2% (13), leading to a poor prognosis of patients. Therefore, it is important to determine the factors affecting PFS in chordoma patients to better guide clinical treatment. AL-Mefty classification, the extent of resection, PDGFR-β, TGF-α, Ki-67, and SNF5 have been reported to affect the prognosis of chordoma patients (14–16). In addition, one previous study established a nomogram to predict the prognosis of chordoma patients according to the clinical characteristics and immunohistochemistry, including the extent of resection, E-cadherin, Ki-67, and VEGFA (12). However, the effect of PALB2 on the prognosis of chordomas has not been previously reported. Our study is the first to show that PALB2 expression is associated with chordoma recurrence, besides, we established a nomogram comprising PALB2 and clinical features for predicting the PFS of chordoma.

Our data confirmed that the extent of resection was significantly affecting the prognosis of patients, consistent with the previous studies (17). Moreover, we found that tumor pathology and Al-Mefty were correlated with PFS. Thus, in clinical management, for conventional chordoma patients with high PALB2 and high Al-Mefty classification, total resection and/or postoperative proton therapy, and close monitoring should be recommended to decrease the potential high recurrent rate. Intraoperative MRI and navigation may be helpful to achieve total resection (18).

Previous studies suggested that PALB2 served as a potential tumor suppressor gene, and its mutation led to increased susceptibility to breast cancer (19). However, our study found that high PALB2 expression patients had an adverse prognosis, and knockdown PALB2 inhibited the proliferation, migration, and invasion of chordoma cells. Previous studies of other tumors mainly focus on PALB2 mutations at the genome level rather than the expression of PALB2 (19, 20). However, few PALB2 mutations were found in large chordoma genome sequencing (21), suggesting that the role of PALB2 mutations reported in other tumors may not be applicable in chordomas. To further understand the effect of PALB2 expression on prognosis, we performed a pan-cancer analysis of the relationships between PALB mRNA level and prognosis in 33 tumor types. The results showed that PALB2 was highly expressed in various tumors and a high PALB2 level was associated with a poor prognosis, which supports our findings. In addition, KEGG enrichment analysis of PALB2-related genes revealed that PALB may promote tumor malignant phenotype *via* regulation of cell cycle, autophagy, and protein degradation.

The function of PALB2 in chordomas has not been reported. Our study is the first to propose high PALB2 indicates an adverse prognosis in chordoma. The mutation of PALB2 is mainly reported to influence DNA homologous recombination repair in other tumors, especially breast cancer (7). While few PALB2 mutations or copy number variation was found in whole-genome sequencing in chordoma (21). This suggested that PALB2 may function through different mechanisms in chordomas. According to previous reports, the homodimerization, phosphorylation, and ubiquitylation of different protein domains and modifications could regulate the PALB2 function (22, 23). Thus, the observed differences in PALB2 expression levels in chordoma might be due to the regulation of transcription and translation. As for the possible downstream mechanisms of PALB2 in chordomas, we hypothesize that PALB2 may influence cell cycle and autophagy based on our analysis in other tumors. Interestingly, PALB2 was reported to restrict the homologous recombination in the S/G2 phase of cell cycle (24). We are currently verifying the above hypothesis and will explore the potential mechanism of PALB2 in chordoma using RNA sequencing.

Interestingly, one recent Phase III clinical trial of non-small cell lung cancer demonstrated that patients with high PALB2 expression were more sensitive to cisplatin - docetaxel chemotherapy (25), suggesting PALB2 as a promising biomarker for the identification of chemotherapy-sensitive patients. Given the high resistance of chordomas to chemotherapy, PALB2 may aid in identifying potential chemotherapy-sensitive chordomas, and further studies are highly needed.

In conclusion, our results reveal that high PALB2 expression indicates a poor prognosis of chordoma patients and promotes the malignant phenotype of chordoma cells *in vitro*.

## Conclusion

In summary, our results reveal that high PALB2 expression indicates a poor prognosis of chordoma patients and promotes the malignant phenotypes of chordoma cells *in vitro*.

## Data availability statement

The original contributions presented in the study are included in the article/**Supplementary Material**. Further inquiries can be directed to the corresponding author.

## Ethics statement

The studies involving human participants were reviewed and approved by Ethics Committee of Beijing Tiantan Hospital. The patients/participants provided their written informed consent to participate in this study.

## Author contributions

YX analyzed the data and carried out experiments. YX and ML wrote the main manuscript text. YS prepared **Figures 1** and **2**. TM and JB collected clinical information on patients. YZ designed and directed the entire study. All authors contributed to the article and approved the submitted version.

## Funding

This research was funded by the National Natural Science Foundation of China (81771489).

## Conflict of interest

The authors declare that the research was conducted in the absence of any commercial or financial relationships that could be construed as a potential conflict of interest.

## Publisher’s note

All claims expressed in this article are solely those of the authors and do not necessarily represent those of their affiliated organizations, or those of the publisher, the editors and the reviewers. Any product that may be evaluated in this article, or claim that may be made by its manufacturer, is not guaranteed or endorsed by the publisher.
