# Supplementary figures and images for: PALB2 as a factor to predict the prognosis of patients with skull base chordoma
Source: Front Oncol. 2022 Sep 8;12:996892. doi: 10.3389/fonc.2022.996892 (PMC9493133; doi:10.3389/fonc.2022.996892)

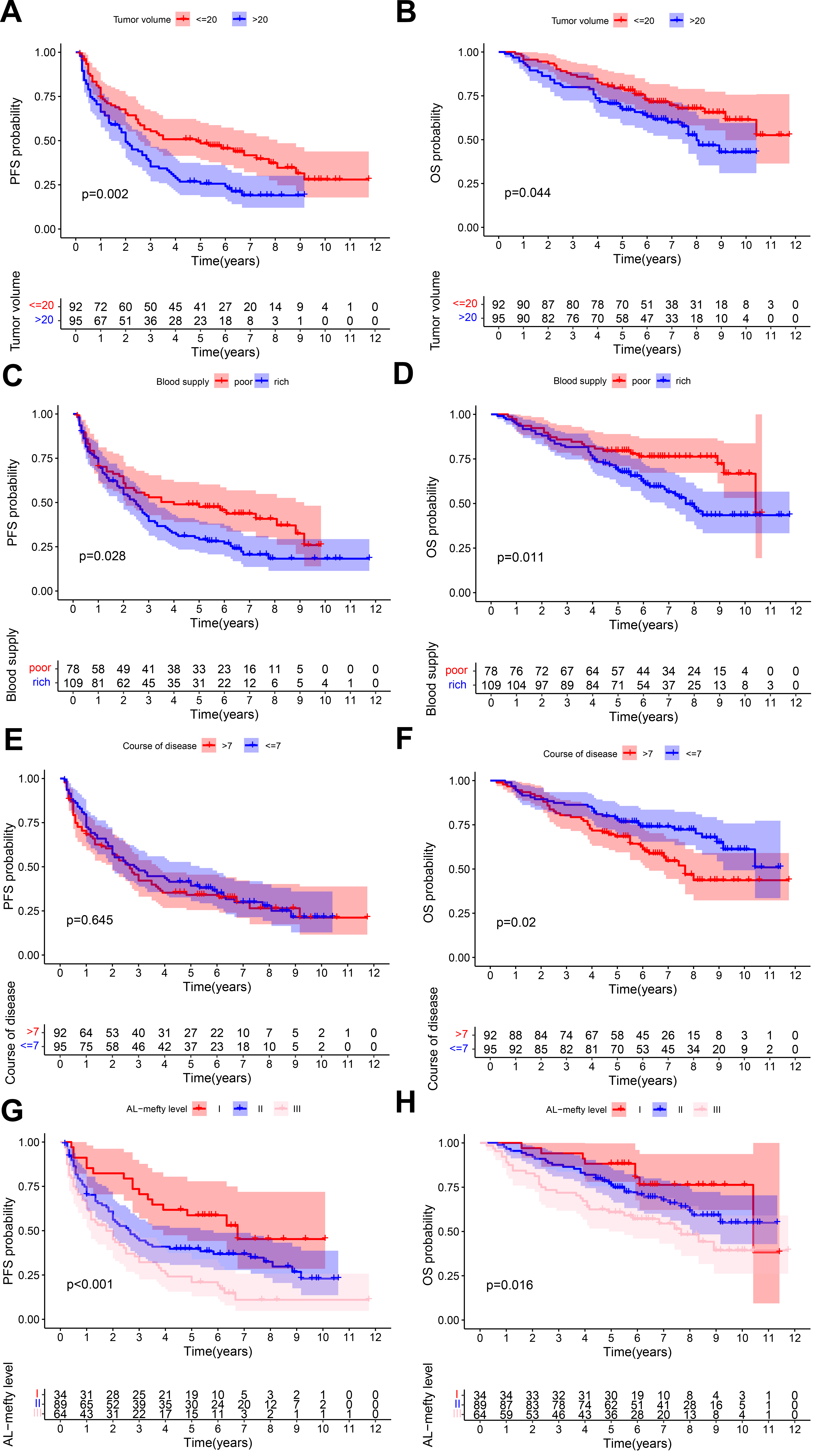

Supplement: Supplementary file 1 [file Image_1.tif]

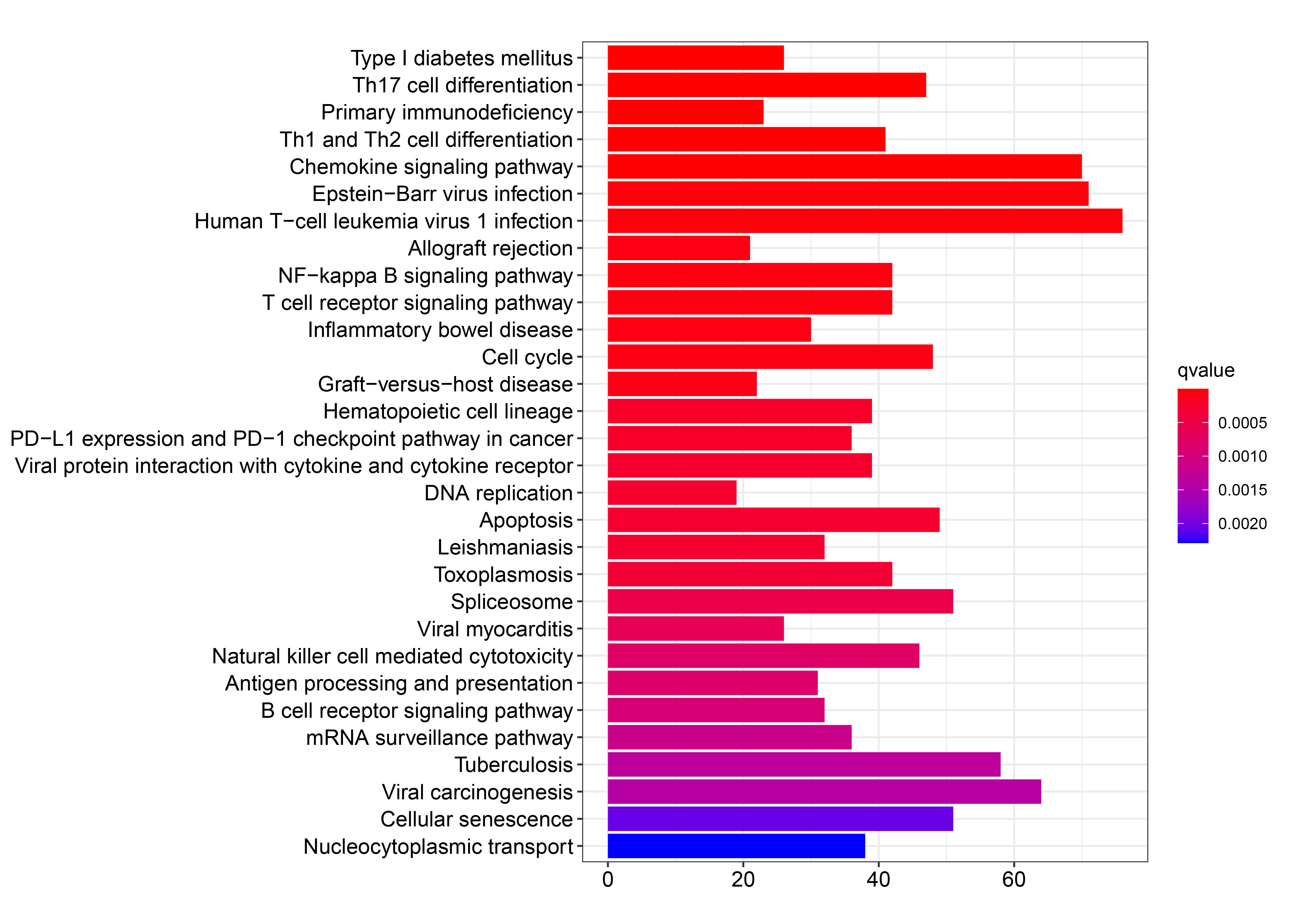

Supplement: Supplementary file 2 [file Image_2.tif]
